# Supplementary material for: GIRK Channel Loss of Function Increases Dendritic Excitability in a Mouse Model of GNB1 Encephalopathy
Source: bioRxiv. 2026 Aug 13:2026.08.08.743706. Preprint. [Version 1] doi: 10.64898/2026.08.08.743706 (PMC13640669; doi:10.64898/2026.08.08.743706)
Supplement: Supplementary file 1 [file NIHPP2026.08.08.743706v1-supplement-1.pdf]

bioRxiv preprint doi: https://doi.org/10.64898/2026.08.08.743706; this version posted August 13, 2026. The copyright holder for this preprint (which was not certified by peer review) is the author/funder, who has granted bioRxiv a license to display the preprint in perpetuity. It is made available under a CC-BY 4.0 International license.

Supplementary Information

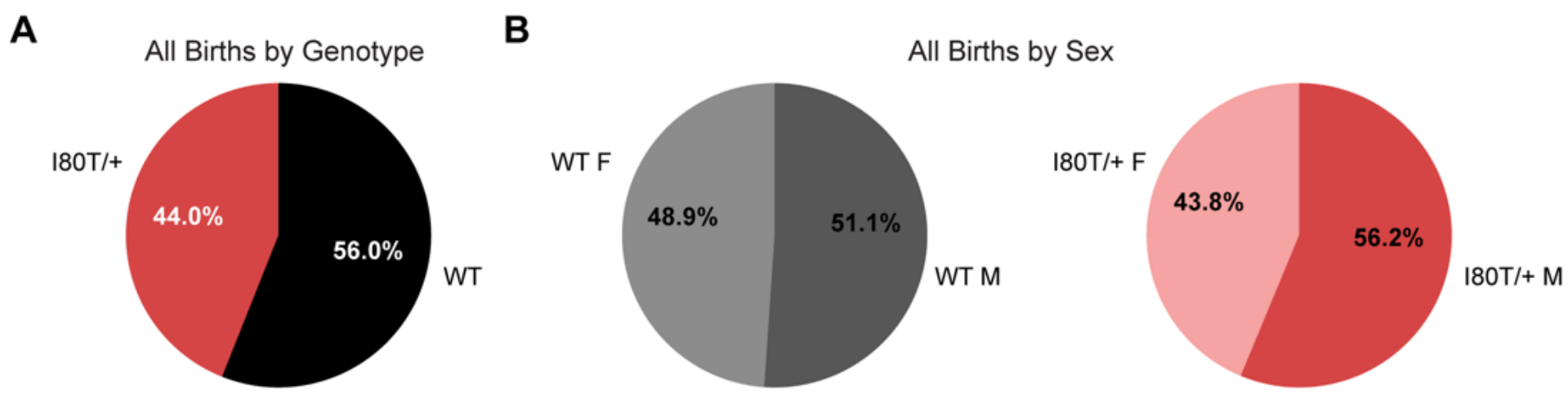

Supplementary Figure 1.
A, Pie chart shows the percentage of offspring of each genotype obtained from breeding heterozygous *Gnb1*$^{I80T/+}$ and WT mice. ***B***, Pie charts show percentage of male and female offspring born of either WT (left) or *Gnb1*$^{I80T/+}$ (right) genotype.

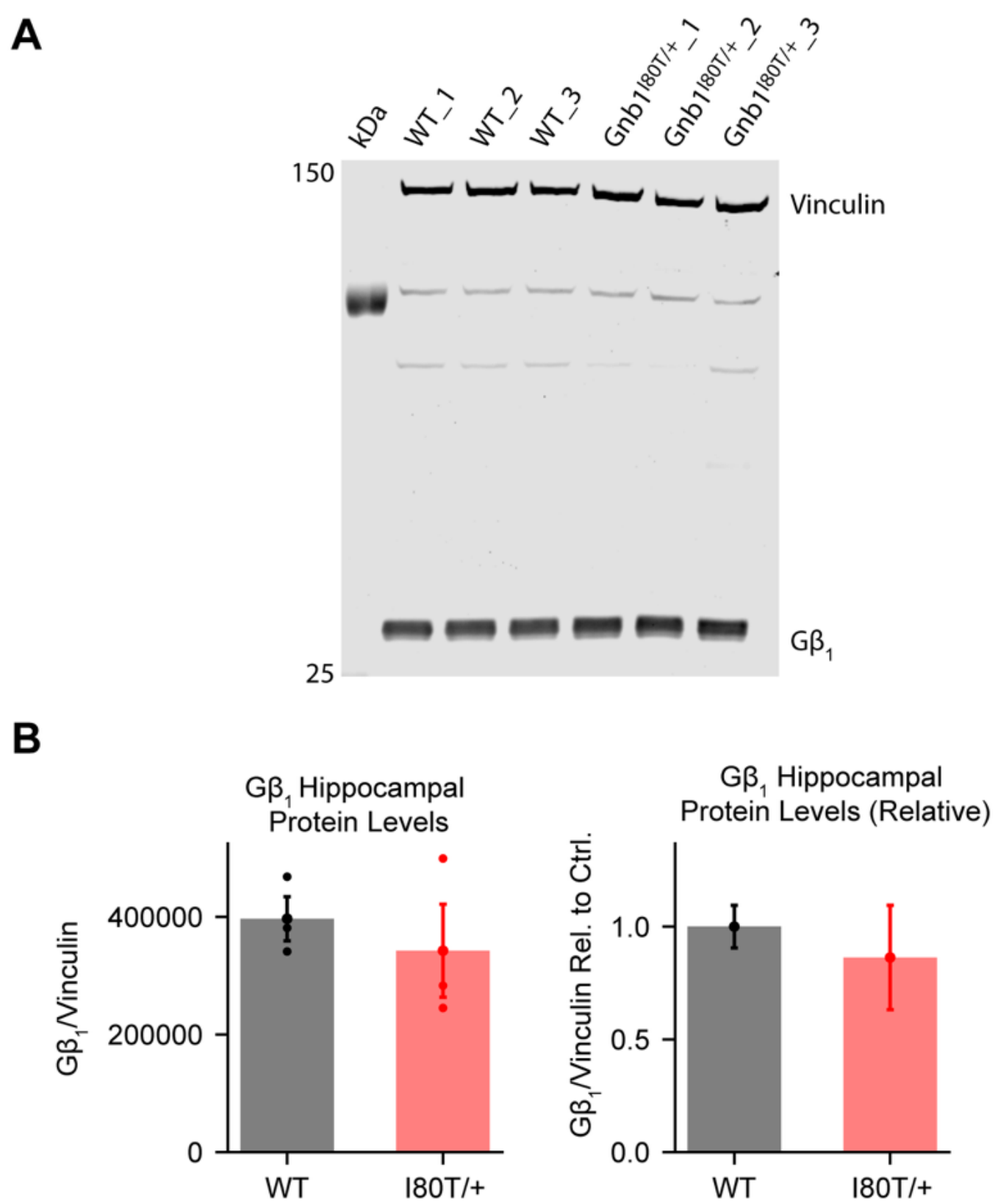

Supplementary Figure 2.
A, Western blot analysis of hippocampal protein lysates isolated from wild-type (WT) and *Gnb1*$^{I80T/+}$ mice with antibodies against $G\beta_1$ and Vinculin, used as a loading control (see Methods). ***B***, Left: $G\beta_1$ protein levels from experiments in ***A*** are quantified as a ratio relative to Vinculin. WT (n = 3): 396666.67 ± 37489.26; *Gnb1*$^{I80T/+}$ (n = 3): 342333.33 ± 79097.69 (U = 6, p = 0.7000). Right: Same as left, with protein ratios normalized to the average levels detected in WT samples. WT: 1.000 ± 0.095; *Gnb1*$^{I80T/+}$: 0.863 ± 0.231 (U = 6, p = 0.7000). Data represented as mean ± SEM. Statistics reflect Mann-Whitney U-tests.

bioRxiv preprint doi: https://doi.org/10.64898/2026.08.08.743706; this version posted August 13, 2026. The copyright holder for this preprint (which was not certified by peer review) is the author/funder, who has granted bioRxiv a license to display the preprint in perpetuity. It is made available under a CC-BY 4.0 International license.

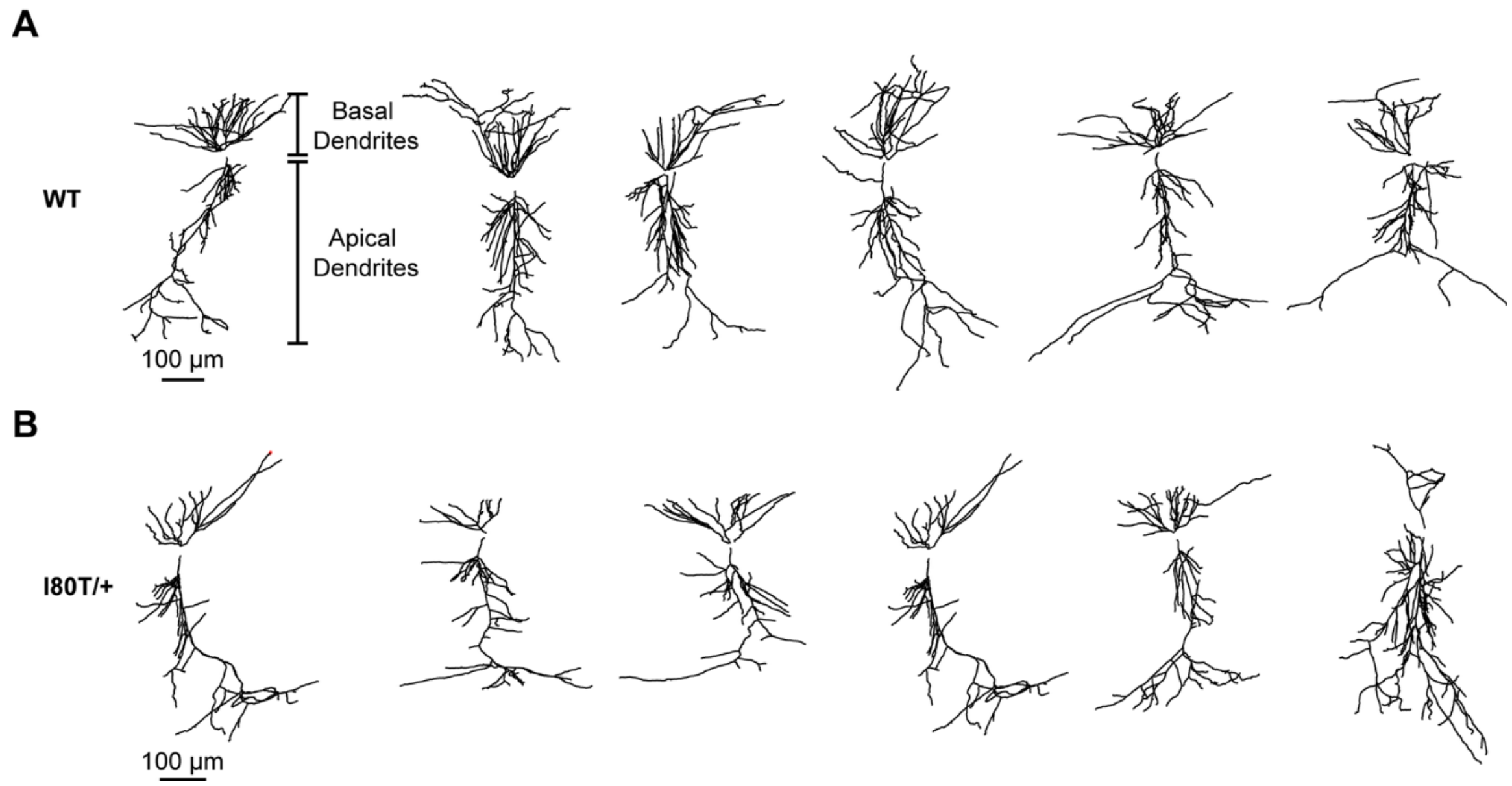

Supplementary Figure 3.
A, Representative histological reconstructions of wild-type (WT) neuron morphologies. ***B***, Same as ***A*** for *Gnb1*$^{I80T/+}$ neurons.

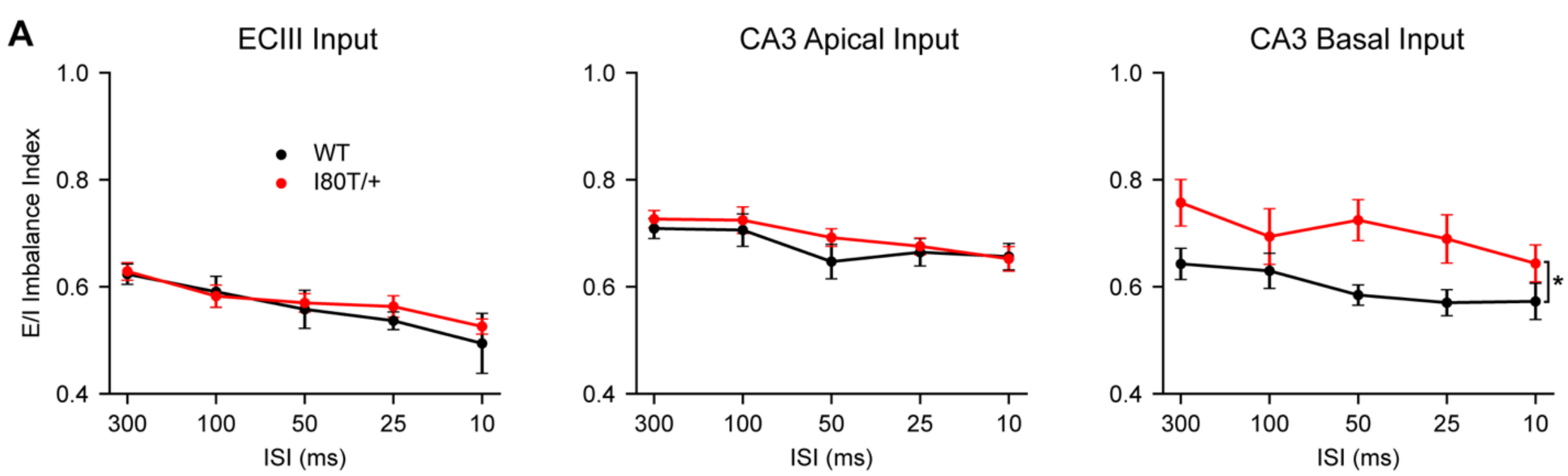

Supplementary Figure 4.
A, The ratio of excitation to inhibition is quantified as (EPSP/(EPSP + IPSP) (see Methods) from responses to stimulation of three input pathways (see Methods). Left: ECIII (WT: n = 12–15, *Gnb1*$^{I80T/+}$: n = 11–13 neurons). Type III ANOVA; main effect of genotype: $F(1, 73.5) = 0.047$, $p = 0.8290$; genotype × ISI interaction effect: $F(4, 105.1) = 0.116$, $p = 0.9767$. Center: CA3 Apical (WT: n = 12–14, *Gnb1*$^{I80T/+}$: n = 10–12). Type III ANOVA; main effect of genotype: $F(1, 65.8) = 0.264$, $p = 0.6093$; genotype × ISI interaction effect: $F(4, 94.0) = 0.542$, $p = 0.7054$. Right: CA3 Basal (WT: n = 11–12, *Gnb1*$^{I80T/+}$: n = 10). Type III ANOVA; main effect of genotype: $F(1, 20.1) = 6.551$, $p = 0.0187$; genotype × ISI interaction effect: $F(4, 79.1) = 0.911$, $p = 0.4620$. All data represented as mean ± SEM. Statistics were performed using linear mixed effects models (see Methods). Asterisks with brackets indicate main effects with $p < 0.05$.
